# Comparison of three different zeolites to activate peroxymonosulfate for the degradation of the pharmaceutical ciprofloxacin in water

**DOI:** 10.1007/s11356-025-35994-4

**Published:** 2025-02-28

**Authors:** Efraím A. Serna-Galvis, Carlos Mendoza-Merlano, Johana Arboleda-Echavarría, Ricardo A. Torres-Palma, Adriana Echavarría-Isaza

**Affiliations:** 1 Grupo de Investigación en Remediación Ambiental y Biocatálisis (GIRAB), Instituto de Química, Facultad de Ciencias Exactas y Naturales, Universidad de Antioquia UdeA, Calle 70 # 52-21, Medellín, Colombia; 2 Grupo de Catalizadores y Adsorbentes (CATALAD), Instituto de Química, Facultad de Ciencias Exactas y Naturales, Universidad de Antioquia UdeA, Calle 70 # 52-21, Medellín, Colombia; 3 Escuela de Microbiología, Universidad de Antioquia UdeA, Calle 70 # 52-21, Medellín, Colombia

**Keywords:** Aluminosilicate, Non-radical pathway, PMS activation, Pollutants degradation, Water treatment

## Abstract

Zeolites are typically used as adsorbents for the removal of organic pollutants from water but recently are gaining attention as catalysts for the activation of persulfates toward contaminants degradation. In this work, the capability of a zeolite Y (FAU-type) and two zeolites beta (BEA-type) to activate peroxymonosulfate (PMS) toward the degradation of one representative pollutant of a pharmaceutical nature (i.e., ciprofloxacin) was tested and compared. Initially, the characterization of the considered zeolites was carried out, evidencing that they had different Si/Al, surface area, and basicity. Then, the main degradation pathway involved in the target pollutant degradation was determined and the activating ability of three zeolites was compared. It was found that among the three tested materials, zeolite Y had the highest activating capability toward PMS for ciprofloxacin degradation (showing ~ 90% degradation after 10 min of treatment). The synergy (*S*) of the systems followed the order: zeolites beta/PMS (*S*, 0.5–1.4) < zeolite Y/PMS (*S*, 3.9), revealing that the Si/Al ratio has a determinant role in the zeolite/peroxymonosulfate combination, being convenient lower values of such a ratio. In the most adequate combination (i.e., zeolite Y/PMS), the pharmaceutical was attacked by singlet oxygen (coming from the PMS activation by the zeolite via basic sites), which modified ciprofloxacin on its piperazyl ring, producing two intermediates. Theoretical analyses based on the structure suggested that the two intermediates have low toxicity against mammals. Additionally, experimental tests showed that the zeolite Y/PMS process led to a resultant solution without antimicrobial activity against *S. aureus*. Finally, it can be mentioned that ZY/PMS was used to deal with ciprofloxacin in synthetic hospital wastewater, achieving ~ 40% pollutant elimination after 60 min of treatment.

**Supplementary Information:**

The online version contains supplementary material available at 10.1007/s11356-025-35994-4.

## Introduction

Minerals such as zeolites are gaining attention as a new approach for the activation of peroxides to produce reactive oxygen species (ROS), which can degrade organic pollutants in water. In fact, zeolite-based systems can overcome some limitations of other oxidative processes such as the high costs associated with the need for specialized equipment (e.g., ultrasound) or energy sources (e.g., UVC lamps) (Li et al. 2019; Kong et al. 2021; Xu et al. 2021; Xiao et al. 2021; Serna-Galvis et al. 2023a). Interestingly, minerals can be reused easily by several cycles retaining high degradation efficiencies (Li et al. 2019; Kong et al. 2021; Serna-Galvis et al. 2023a).

On the other hand, organic pollutants (e.g., pharmaceuticals) in water are a worrying problem worldwide. For instance, antibiotics are beneficial in the treatment of infections saving millions of people, but at the same time, these pharmaceuticals have also emerged as environmental contaminants. Indeed, the continuous input of antibiotics into the environment is considered a source/promoter of antimicrobial resistance proliferation (Patel et al. 2019). Nowadays, it is well-known that conventional processes applied in municipal wastewater treatment plants (WWTPs) are not capable of degrading most pharmaceuticals (Patel et al. 2019). Consequently, these organic pollutants reach the environment. In fact, pharmaceuticals (e.g., antibiotics) are concerning pollutants in groundwater (Bunting et al. 2021). Hence, treatment processes efficient for the abatement of such contaminants are required.

Advanced oxidation processes (AOPs, i.e., processes based on the generation and use of ROS) are valuable for degrading recalcitrant pollutants in water (Patel et al. 2019). Among AOPs, the heterogeneous processes are widely used for organic pollutants degradation due to their versatility to involve, in photocatalytic and non-photocatalytic systems, solid materials from diverse nature (e.g., iron oxides, ZnO, Co-based zeolite imidazolate frameworks-ZIF, and non-modified zeolites) (Sheikhmohammadi et al. 2021, 2023, 2024; Serna-Galvis et al. 2023a; Tang et al. 2024). For instance, ZIF nanostructures have been successfully used for treating antibiotics in water by photocatalytic systems (Sheikhmohammadi et al. 2024).

On the other hand, the AOPs based on the activation of persulfates (i.e., peroxydisulfate or peroxymonosulfate), using particular heterogeneous catalysts to obtain high degrading activity and oxidation rates, are topics of increasing interest (Tang et al. 2024). Also, the elimination of organic pollutants with ROS from the activation of peroxymonosulfate (PMS) is gaining attention due to the high degradation efficiency of such systems and operational easiness (Sbardella et al. 2020; Ushani et al. 2020; Wang et al. 2021; Xiao et al. 2021). Indeed, PMS activation using zeolites loaded with transition metals, producing ROS for degrading organic pollutants in water, is common, and these materials typically evolve electron transfer mechanisms to produce radical species (Fu et al. 2020; Elmaadawy et al. 2021). However, non-modified zeolites are interesting catalysts because such materials have shown great potentiality to activate PMS through the promotion of non-radical pathways, even leading to selective degradation of organic pollutants in complex matrices (Serna-Galvis et al. 2023a).

Zeolites are aluminosilicates, which have low-cost extraction or synthesis (Li et al. 2019; Khaleque et al. 2020). Zeolites and aluminosilicates have been typically utilized as supporting materials for different classes of catalysts (Fu et al. 2020; Liu et al. 2020; Chen et al. 2022; Wang et al. 2022). Also, zeolite-based materials, such as ZIF nanocomposites, can be used as photocatalysts in AOPs for antibiotics degradation (Sheikhmohammadi et al. 2024). Furthermore, the testing of zeolites or other aluminosilicates to activate inorganic peroxides is increasing. Indeed, a kaolinite has been used for activating PMS to degrade atrazine in water samples, and such work reports the PMS activation, producing radical species (Li et al. 2019). Recently, the zeolite 4A was tested for the activation of PMS, informing the generation of the non-radical pathway (e.g., the action of singlet oxygen) to degrade organic pollutants (Serna-Galvis et al. 2023a). However, the comparison of diverse zeolites (i.e., the effect of zeolite architecture/type) and the role of the Si/Al ratio on the activation of PMS has not been explored. These topics are presented herein.

This work aimed to study the effect of zeolite type and the role of the Si/Al ratio to activate PMS (via a non-photocatalytic process) toward the degradation of a representative organic pollutant. Hence, the zeolite Y (ZY) and a zeolite beta (ZB) previously synthesized in our research team (Manrique et al. 2016; Mendoza and Echavarría 2022), plus a commercial BEA-type zeolite (i.e., CP814C* (Zeolyst International 2015)), were considered. Initially, a strategic characterization of the considered zeolites was carried out. Then, the activating ability of the considered zeolites was compared. Afterward, ZY (which presented the highest activating capability) was used to determine the main pathways involved in the target pollutant degradation. Also, for the zeolite/PMS system having the best activating performance, the primary transformations induced on the pollutant, the antimicrobial activity evolution, and the matrix effects were assessed. It is important to mention that, based on the chemical structure of the primary by-products coming from pollutant degradation, theoretical calculations about antibiotic activity, toxicity, and biodegradability were carried out. Additionally, the molecular regions on CIP more susceptible to attacks by the generated ROS were determined using the net charge on the atoms, which were consistent with the by-products observed experimentally.

## Materials and methods

### Reagents

The antibiotic ciprofloxacin (CIP) was selected as a probe pollutant considering that this pharmaceutical is able to induce both chronic and acute harmful effects on natural fauna and flora. CIP is commonly found in environmental water and the effluents of hospitals and municipal wastewater treatment plants (Verlicchi et al. 2015; Oliveira et al. 2018; Botero-Coy et al. 2018; Patel et al. 2019). This antibiotic can promote the development and proliferation of antibiotic-resistant bacteria (Sidhu et al. 2021). Furthermore, CIP has been included in the European watch list of substances for Union-wide monitoring in water policy (Decision 2020/1161) (Decision 2020/1161/EU 2020). Besides, to provide structural diversity and different Si/Al ratios, three representative zeolites were chosen. Thereby, a zeolite Y (FAU-type) and two BEA-type zeolites were used herein.

ZY and ZB were previously synthesized and reported by our research team (Manrique et al. 2016; Mendoza and Echavarría 2022). CP814C* was obtained from Zeolyts International. CIP hydrochloride was provided by Laproff Laboratories. Oxone® (KHSO_5_, 0.5KHSO_4_, 0.5K_2_SO_4_) was purchased from Sigma. Ammonium chloride and calcium chloride dihydrate were obtained from PanReac. Acetonitrile, methanol, methyl orange, potassium chloride, sodium azide, sodium chloride, sodium hydroxide, sodium dihydrogen phosphate, sodium sulfate, sulfuric acid, and urea were obtained from Merck. Formic acid was purchased from Carlo Erba.

### Analyses

The confirmation of the crystalline phase of the zeolites was done using the X-ray diffraction (XRD) technique in a PANalytical Empyrean X-ray 2012 powder diffractometer, operated in reflection transmission spinner geometry with Cu-Kα radiation at 40 mA and 45 kV, and in the reflection mode between 3 and 50° (2*θ* angle). The Brunauer, Emmett, and Teller (BET) method was utilized to calculate the surface area, and the nitrogen adsorption–desorption isotherm was measured in a Micromeritics ASAP 2020 apparatus at 77 K. Before the BET analysis, the zeolites were degassed under high vacuum conditions for 8 h at 350 °C.

The basicity of the considered zeolites was determined through analyses of temperature-programmed desorption (TPD) of CO_2_, employing a Chemisorb 2720 by Micromeritics. A sample (300 mg) was placed into the quartz reactor, degassed to 500 °C for 1 h, and saturated with CO_2_ pulse at room temperature. The desorbed process was carried out from 30 to 1000 °C at 10 °C min^-1^ in a helium flow of 30 mL min^-1^ and the CO_2_ signal was monitored by a mass spectrometer.

The best material for the PMS activation toward the CIP degradation was characterized (before and after the reaction) by X-ray photoelectron spectroscopy (XPS) using a PHOIBOS 150 1D-DLD analyzer and monochromatic Al-K_α_ radiation (1487 eV) set up at 100 W, with a pass energy of 20 eV and step size of 0.1 eV. A low-energy electron flood gun (20 mA emission current and 3 eV cathode voltage) was used for the charge compensation. To calibrate the energy scale, an adventitious C1s core level line was utilized. A Gaussian–Lorentzian blend (GL30%) and a Shirley-type background subtraction were used to analyze the spectra.

The evolution of CIP was followed by utilizing liquid chromatography. A Thermo Scientific (Ultimate 3000 UHPLC) apparatus, having a diode array detector and an Acclaim™ 120 column (C18, 5 µm, 4.6 × 150 mm), was employed. A 15/85 mixture of acetonitrile/formic acid (10 mmol L^−1^) at 1.0 mL min^−1^ as mobile phase in isocratic mode, 20 μL of injection volume, and 278 nm as detection wavelength were the analytical conditions.

For the theoretical calculations about the molecular regions on CIP more susceptible to attacks by ROS, the CIP structure was optimized using the polarized continuum model and water dielectric constant with a B3LYP density functional (Raghavachari 2001; Tomasi et al. 2005), and a 6 − 31 + *g*(*d*) basis set was utilized for the calculations, as implemented in Gaussian 03 Rev.E01 software to obtain the net charge on the atoms from the natural bonding orbitals (NBO) analyses.

The primary degradation products coming from the treatment of CIP by the zeolite/PMS systems were elucidated using an HPLC Agilent 1200 series, coupled with an Agilent LC/MSD VL SQ mass spectrometer. The mobile phase and column were the same used for following the CIP evolution. Moreover, the sample (10 µL) was injected, and the mass spectrometer detection was performed in the positive ion mode.

To provide insights into practical applications of the catalytic process, the reuse test, for the zeolite after multiple cycles of PMS activation, was carried out following the procedure detailed in the reference (Serna-Galvis et al. 2023a). The antimicrobial activity was determined through the inhibition zone method (i.e., Kirby-Bauer method), using *Staphylococcus aureus* ATCC 6538 as indicator bacteria. This microorganism was used because it is an important human pathogen able to develop resistance to diverse antibiotic classes (Szabó et al. 2016). These experimental tests were also combined with antimicrobial biological activity predictions of CIP and its primary degradation products, which were obtained using the PASS software (free online version), having structure–activity relationships as conceptual bases (W2D Team - PharmaExpert 2021). The chemical structures of CIP and its by-products were individually uploaded (in the SMILE format) to the software. Subsequently, the values of the probability of biological activities (Pa) for the tried substances are outputted by the PASS software.

In addition to the analyses related to the antibiotic activity, the biodegradability and biological toxicity of CIP and its primary degradation products were also outlined by using the BiodegPred tool, which provides a prognosis about the toxicity and the chance of a target substance being catabolized in the biosphere (Garcia-Martin et al. 2020b, a). In the BiodegPred tool, the biodegradability criterion is constructed on a test database from the Ministry of International Trade and Industry-Japan (NITE), which considers that the target substance is inoculated and incubated with 30 mg L^−1^ of activated sludge, following the biological oxygen demand (BOD) for 28 days. If the BOD is > 60% of the theoretical oxygen demand, the target substance is considered “ready biodegradable”; otherwise, the substance is named “non-ready biodegradable”.

The toxicity criterion in BiodegPred is based on mammalian oral toxicity (usually taken as a proxy for human toxicity), which uses the Pesticide Properties Data Base (PPDB) from the University of Hertfordshire. The toxicity classification of PPDB considers the acute oral LD_50_ in mammals; i.e., those substances presenting LD_50_ > 2000 mg/kg have “low toxicity,” and compounds with LD_50_ ≤ 2000 mg/kg exhibit “high toxicity” (Garcia-Martin et al. 2020b). Thus, to carry out the predictions of biological toxicity and biodegradability, the chemical structures of CIP and its by-products, in SMILE formats, were individually uploaded on the free online software BiodegPred. This tool generates scores for the biodegradability or toxicity parameters, allowing us to discriminate between two categories: “low toxicity” and “high toxicity” for the toxicity; or “ready biodegradable” and “non-ready biodegradable” for the biodegradability parameter. The two categories are separated by a reference score (score = 0). Thereby, the tested substance lies in “high toxicity” for the toxicity parameter and the “non-ready biodegradable” category for the biodegradability parameter if the scores are higher than zero. In turn, scores lower than zero indicate that the target substance belongs to the “low toxicity” for the toxicity parameter and the “ready biodegradable” category for the biodegradability parameter (Garcia-Martin et al. 2020b).

### Reaction system

The activation of PMS by the zeolites was carried out in a beaker under a magnetic stirring system. A solution (50 mL) of the target contaminant was used, and the PMS and zeolite were added at 500 µmol L^−1^ and 0.2 g L^−1^, respectively. The dose of zeolite and PMS concentration were selected taking into account some previous works, which have utilized PMS in the range of 50–1000 µmol L^−1^ and 0.2–1.0 g L^−1^ for the solid materials as feasible amounts for a proper activation process (Guan et al. 2017; Li et al. 2019; Fu et al. 2020; Sun et al. 2020; Dai et al. 2021; Wang et al. 2022; Serna-Galvis et al. 2023a). It is important to mention that the experimental procedures were carried out at least by duplicate, and the experimental results are presented as the average value with its corresponding standard deviation.

## Results and discussion

### Characterization of the zeolites

**Fig. 1:**
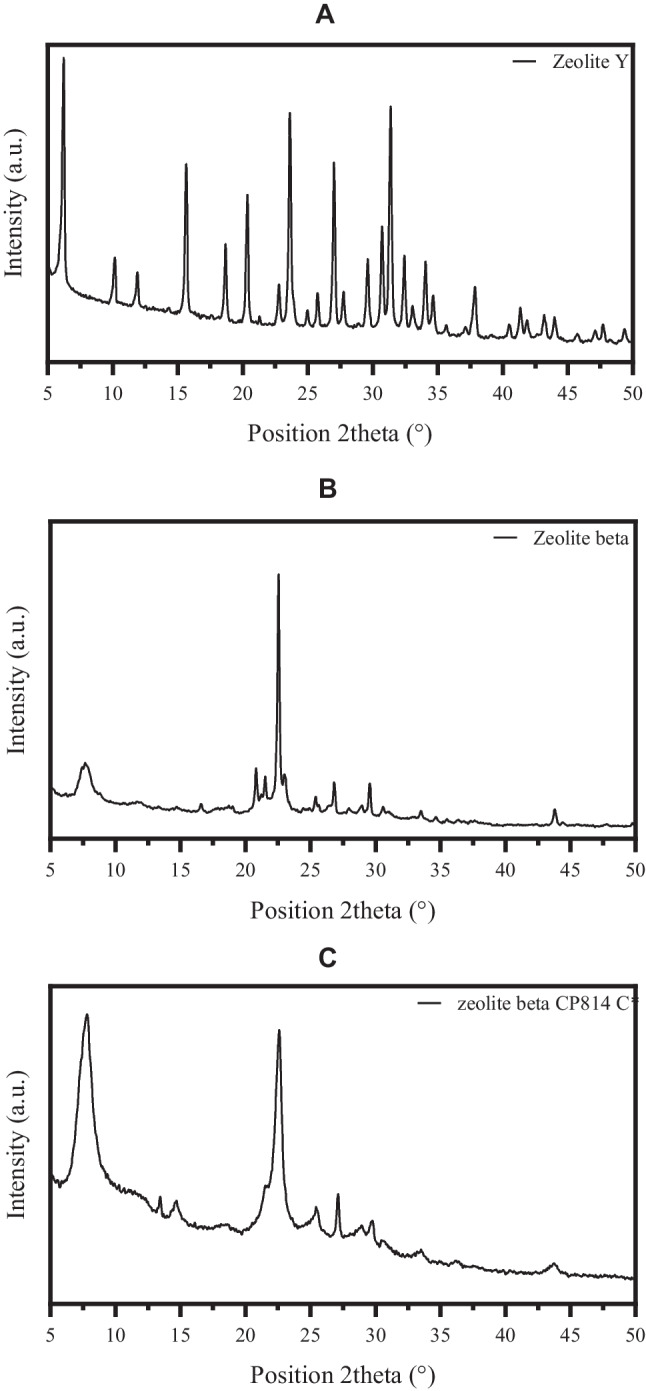
X-ray diffraction spectra (XRD) of the considered zeolites. **A** XRD for ZY, **B** XRD for ZB, and **C** XRD for CP814C*

To confirm the crystalline structure, the XRD analyses of the considered zeolites were carried out (Fig. 1). The results showed the typical diffraction pattern for zeolites Y and BEA types. For instance, peaks at very representative Bragg angles (2*θ*) positions of ZY, such as 6.169, 10.083, 11.829, and 15.566°, are observed in Fig. 1A for the typical Y structure (ICSD 94779) (Fowkes et al. 2002). In the case of ZB, peaks at 2*θ* equal to 7.847, 22.430, 25.360, and 27.183° were found (Fig. 1B), which allowed to verify the presence of the BEA structure (ICSD 153253) (Martínez-Iñesta et al. 2005). Also, CP814C* exhibited the typical peaks for a BEA structure (Fig. 1C) as seen for ZB (Fig. 1B) but with the signals shifted to a high angle due to the elevated Si/Al ratio (Rajaei et al. 2021).

In addition to the XRD analyses, the surface area of the considered zeolites is determined and presented in Table 1. It can be noted that ZY, ZB, and CP814C* had large surface areas (324–919 m^2^/g), which are indicative of their highly porous nature, which is a common feature among zeolites with three-dimensional micropore systems (Kenvin et al. 2016).

**Table 1:** Relevant characteristics of the tested zeolites

Zeolite	Si/Al* (molar ratio)	S_BET_* (m^2^/g)	CO_2_ adsorbed* (µmol g^−1^)
ZY	2.7	919	9
ZB	8.6	324	23
CP814C*	19	710	16

*Si/Al, silica/alumina ratio, *S*_*BET*_, specific surface area, and CO_2_ adsorbed is an indicator of the basicity of the considered zeolites

Besides the surface area determinations, the basicity of the zeolites was explored using the TPD of CO_2_ (Table 1 and Fig. S1 in the supplementary material). The basicity analyses evidenced that ZB has the highest number of basic sites followed by CPE814C*. It can be noted that for the BEA-type zeolites, the basicity decreases as the Si/Al ratio increases. As the Si/Al ratio increases, the number of basic sites decreases due to the lower presence of = Al-O- and Si–O-Al moieties. However, as the Si/Al ratio increases, the basic sites change the strongness from medium (420 °C) to strong (850 °C) due to lower competition between the sites in the crystal lattice (Li et al. 2022). Also, it can be remarked that ZY, which has a lower Si/Al ratio than ZB, shows weak basic sites that are not present in the BEA-type zeolites (Fig. S1). The CO₂ adsorbed in the supercages of FAU-type zeolite can interact mainly with exchangeable cations. However, in the BEA framework, the probe molecule not only interacts with exchangeable cations but also engages with nearby strong basic oxygens along interconnected channels and pores (Schoonheydt et al. 2012; Otomo et al. 2019).

### Capability of the diverse zeolites to activate PMS for the organic pollutant degradation

**Fig. 2:**
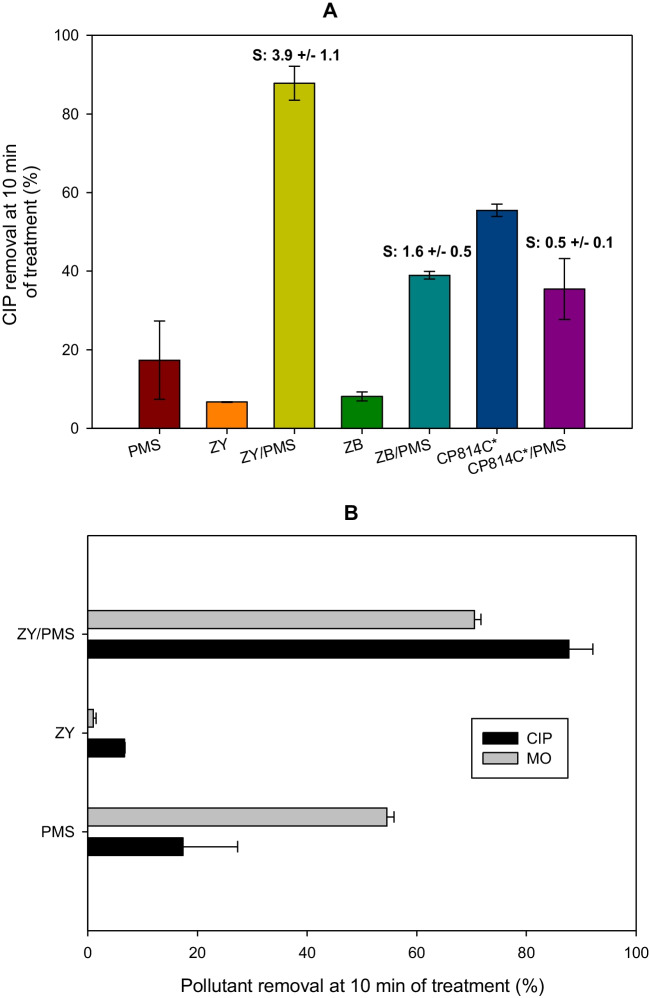
Degradation of organic pollutants by the zeolite/PMS system. **A** Effect of the zeolite type on the CIP degradation. **B** Comparison of CIP and MO degradation by the ZY/PMS combination. *Experimental conditions:* [Zeolite] = 0.2 g L^−1^, [PMS] = 500 µmol L^−1^, and [CIP] = [MO] = 30.6 µmol L^−^.^1^, and initial pH = 5.6. *Note:* The *S* inside **A** is the synergy value for the zeolite/PMS systems, calculated as *S* = CIP removal by zeolite/PMS*/*(CIP by PMS + CIP removal by zeolite)

A FAU-type zeolite (ZY) and two BEA-type zeolites (i.e., ZB and CP814C*) were evaluated, considering that these kinds of zeolites are widely utilized in the conventional process of organic micropollutants adsorption (Jiang et al. 2018). Figure 2 compares the performances of the three zeolites, presenting the CIP removals by PMS and zeolite individually, plus the action of the zeolite/PMS combination. It can be noted that both ZY/PMS and ZB/PMS synergistically degraded CIP (i.e., the removal by the zeolite/PMS system was much higher than the obtained from the arithmetic sum of the PMS and zeolite acting individually). However, the ZY/PMS showed higher synergy and degradation efficiency. From Table 1, it is observed that ZB has higher Si/Al and lower surface area than ZY, which could explain the differences in the synergy and CIP degradation efficiency.

To better study the effect of surface area and Si/Al ratio influence on the treatment of CIP, we considered the case of zeolite CP814C* in detail. As shown in Table 1, this BEA-type zeolite has a surface area close to ZY but a higher Si/Al ratio (i.e., 19). (Fig. 2A). The results showed that the adsorption of the target pollutant on the zeolite CP814C* was significantly increased regarding ZY and ZB. However, the CP814C*/PMS combination was antagonistic (synergy value, S, 0.48). Despite ZY having the highest surface area, the CIP adsorption was lower than on the BEA-type zeolite. The lower CIP adsorption on ZY can be associated with the aluminum content, and consequently, with the zeolite hydrophobicity.

As the Al content decreases (see Table 1), the zeolite is more hydrophobic, thus favoring the organic pollutant-zeolite hydrophobic interactions (i.e., hydrophobic zeolites prevent the pore blockage by water clusters, resulting in more pores available for diffusion and adsorption of pollutants (Jiang et al. 2018)), and thus, the adsorption is higher (Fig. 2A). Furthermore, a competence between CIP and PMS to interact with the zeolite surface could explain the antagonistic results for the CP814C*/PMS combination (Fig. 2A).

On the other hand, it can be noted that as the Si/Al ratio increased, the synergy of the zeolite/PMS combination diminished (Fig. 2A), suggesting a relevant role of this parameter, more than surface area, in the PMS activation process. We should mention that when the Si/Al is lower as in ZY, = Al-O- and Si–O-Al groups (i.e., basic moieties) are present at the zeolite surface at higher levels. As shown in the “Characterization of the zeolites” section, for the two BEA-type zeolites, the basicity decreased as the Si/Al ratio increased. Moreover, the ZY, which has the lowest Si/Al ratio, showed accessible weak basic sites that are not present in the BEA-type zeolites (Table 1 and Fig. S1). Thus, the basic groups (BG^−^) on the surface of the zeolites promote the deprotonation of PMS (Eq. 1), and the deprotonated PMS (SO_5_^2−^) can make a nucleophilic attack to the peroxide oxygen of PMS, resulting in the formation of ROS (Eq. 2) (Serna-Galvis et al. 2023a) that lead to CIP degradation.

1 $${BG}^{-}+{{HSO}_{5}}^{-}\to BGH+{{SO}_{5}}^{2-}$$

2 $${{SO}_{5}}^{2-}+{HSO}_{5}^{-}\to ROS$$

To test the versatility of the ZY/PMS system toward another class of pollutants, the degradation of methyl orange (MO, which is a representative molecule of the azoic dyes family, Fig. S2A (Pandey et al. 2024)) was assessed. The MO removal by ZY/PMS and its corresponding subsystems (ZY and PMS) is presented in Fig. 2B. It can be observed that the catalytic system achieved 71% degradation at 10 min of treatment. In turn, no adsorption of MO on the zeolite was observed, but the PMS alone promoted the direct oxidation of MO (55%). However, the ZY/PMS system led to a higher degradation of the dye (Fig. 2B).

The anionic sulfonate group of the azo-dye experiences electrostatic repulsions with the negatively charged moieties on the ZY surface; then, the MO adsorption is ruled out (Fig. 2B). In the case of PMS, this oxidant directly promotes attacks on the azo group on MO, thus decreasing the dye concentration. Meanwhile, the ZY/PMS combination led to a higher MO degradation due to the action of ROS, whose production is associated with the interaction between the zeolite and peroxymonosulfate (Serna-Galvis et al. 2023a).

When comparing the degradations of MO and CIP by ZY/PMS, we find that the synergy for the CIP degradation was higher (Fig. 2B). This difference is explained by considering the dye has a much higher degradation by the direct action of PMS that decreases the synergy. Then, the results in Fig. 2B suggest that the ZY/PMS combination is more useful/efficient for organic pollutants with low adsorption on the zeolite, low oxidation by PMS, and high reactivity toward the ROS formed by the combination.

### Degradation routes in the treatment of the pollutant by the combination of PMS with zeolite Y

**Fig. 3:**
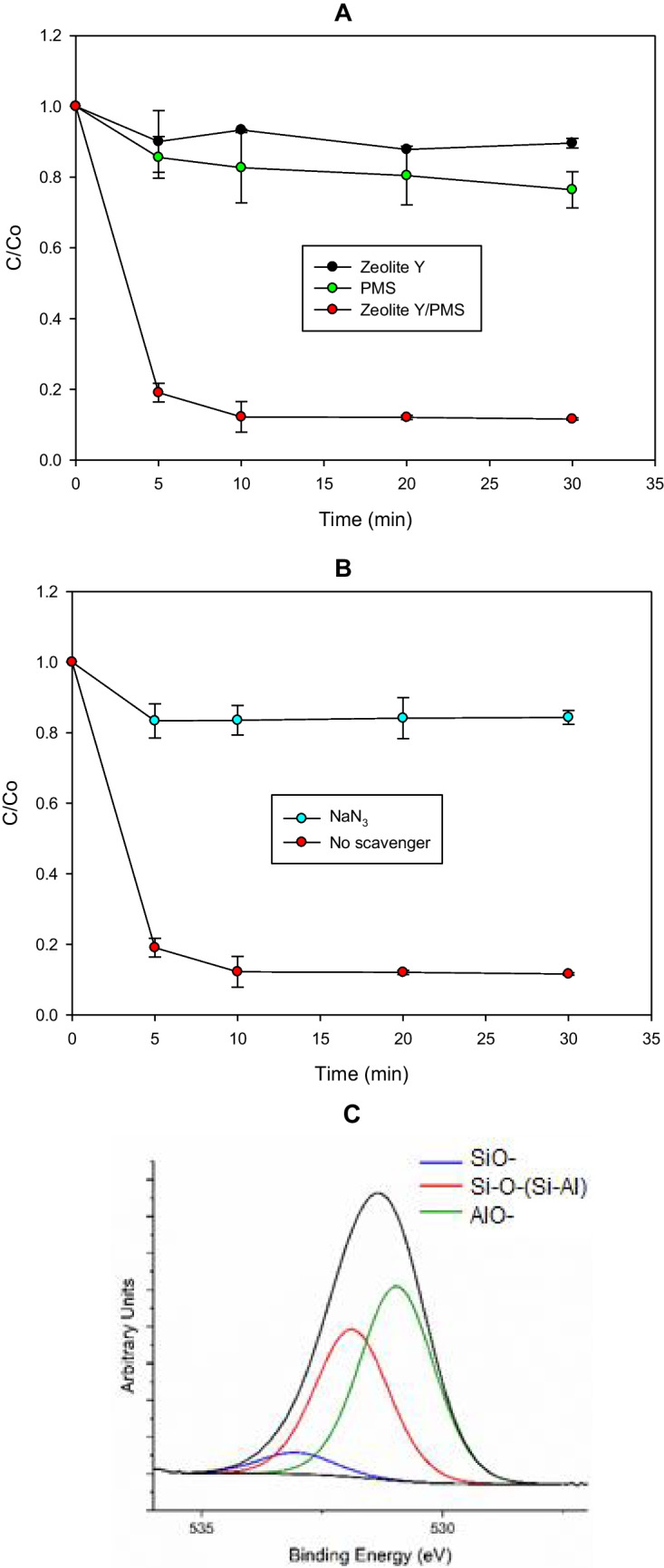
Elucidation of the routes involved in the treatment of CIP by the activation of PMS by ZY. **A** Degradation of CIP by the ZY/PMS system. **B** Effect of a singlet oxygen scavenger. **C** XPS high-resolution spectra for orbital O1s of superficial oxygens on ZY before interaction with PMS. *Experimental conditions:* [Zeolite] = 0.2 g L^−1^, [PMS] = 500 µmol L^−1^, and [CIP] = 30.6 µmol L^−1^, [NaN_3_] = 3060 µmol L^−1^, and initial pH = 5.6

After evidencing the highest capability of the zeolite Y to activate peroxymonosulfate toward the degradation of CIP (Fig. 2A), the ZY/PMS process was considered more deeply. Figure 3A depicts in detail the treatment of the target compound using ZY. The evolution of the target pollutant under the action of PMS and ZY individually and the ZY/PMS combination is presented herein. It can be noted that the PMS alone degraded ~ 18% of CIP. Also, it was found that ZY alone showed low adsorption of the probe pollutant (less than 10% after 10 min of treatment). In contrast, the ZY/PMS combination achieved 88% of CIP removal after 10 min of the process action.

Zeolites are classified according to their Si/Al ratio as high siliceous (Si/Al ratio ≥ 10), intermediate (Si/Al ratio from 2 to 5), and aluminous ones (Si/Al ratio between 1.0 and 1.5) (Cataldo et al. 2021). Zeolites having a high Si/Al ratio are hydrophobic and organophilic materials widely used in adsorption-related applications (Braschi et al. 2010; Cataldo et al. 2021; Feng et al. 2021). Indeed, high-silica zeolites show favorable characteristics for the adsorption of neutral and anionic organic contaminants in aqueous solutions, because such zeolites have a limited number of cations and negative charges around Al sites, and most of the framework structures remain neutral (Jiang et al. 2018). Our ZY is not a hydrophobic zeolite as evidenced by its Si/Al ratio equal to 2.7 (Table 1), which disfavors the adsorption by hydrophobic interactions. Then, the observed adsorption on ZY can be associated with some electrostatic interaction between CIP (which at the experimental pH has positively charged the piperazyl ring, Fig. S2B) and the negative = Al-O^−^ groups on the zeolite Y surface. On the other hand, peroxymonosulfate has a high redox potential (E°= 1.82 V (Kiejza et al. 2021)) and this can directly oxidize organic compounds such as CIP. Therefore, partial removal of the pollutants by direct oxidation was observed in Fig. 3A. In fact, the literature reports that PMS can react with amine moieties as those present in CIP (Zhou et al. 2018; He and Shea 2020).

The ZY/PMS combination led to a synergistic removal of the pollutant (Fig. 3A), which can be associated with the formation of ROS from the interaction of peroxymonosulfate with the zeolite. As the used ZY has no metals such as Fe, Cu, Co, or Mn (Table S1, in the Supplementary material), the PMS activation can be mainly associated with non-radical species. Previous works have also informed that the activation of PMS by zeolites produces non-radical ROS such as singlet oxygen (Eqs. 1 and 3) (Serna-Galvis et al. 2023a).

3 $${{SO}_{5}}^{2-}+{HSO}_{5}^{-}\to {{HSO}_{4}}^{-}+{{SO}_{4}}^{2-}+{}^{1}{O}_{2}$$

**Fig. 4:**
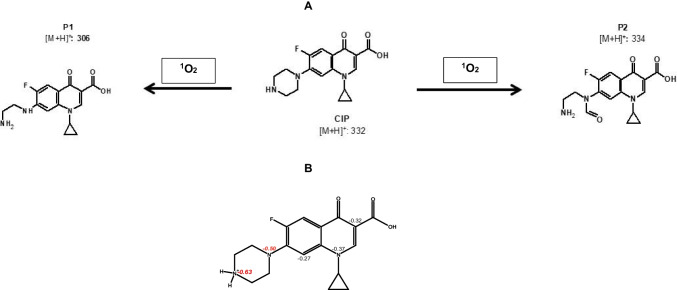
**A** Primary transformations of CIP by the singlet oxygen generated from the ZY/PMS system obtained by LC–MS analyses. **B** Net charge on CIP atoms obtained by the NBO computational analysis

To elucidate the participation of singlet oxygen, the CIP treatment by the ZY/PMS system, in the presence of sodium azide (NaN_3_, which is a scavenger of ^1^O_2_ (Lee et al. 2020; Serna-Galvis et al. 2023a), was performed (Fig. 3B). When NaN_3_ was present in the solution, the degradation of the pollutant was strongly inhibited (only 15% of CIP was degraded after 30 min, Fig. 3B). Despite sodium azide can also directly react with PMS (Lee et al. 2020; Serna-Galvis et al. 2023a), the strong inhibition observed in Fig. 3B indicates the participation of singlet oxygen in the target pollutant degradation. It is important to mention that the formation of only two primary degradation products (Fig. 4A) also supports the action of a non-radical ROS (i.e., ^1^O_2_) because this is more selective than radical species (which can induce the formation of several by-products (Serna-Galvis et al. 2023a)). Also, extra analyses such as electron-paramagnetic resonance (EPR) could be used in future works with zeolites to assist in the identification of ROS types involved in catalytic systems (Tang et al. 2023, 2024).

As mentioned above, the basic groups on ZY may be involved in the PMS activation, Hence, to confirm the activation mechanism proposed in Eqs. 1–3, X-ray photoelectron spectroscopy (XPS) analyses were made to ZY before and after the interaction with PMS (Fig. 3C–D). As the basic groups on the zeolites involve oxygenated moieties, the XPS high-resolution spectra for the superficial oxygen on ZY and their corresponding deconvolution were considered. Signals of the oxygen atoms belonging to three different environments: Al-O^−^ (aluminate anions), Si–O-(Si-Al) bonds, and Si–O^−^ (silanolate groups) are found in Fig. 3C. Moreover, Table S2 presents the quantitative results of the deconvolution of O1s spectra for the basic groups on the ZY surface (i.e., Al-O^−^ and Si–O^−^).

The comparison between basic groups on the ZY surface before and after interaction with PMS (Table S2) revealed a diminution of the Al-O^−^ and Si–O^−^ moieties. This supports the first step of the PMS activation, i.e., acquisition of a proton by the basic sites of ZY from the PMS (Eq. 1). Then, to verify the role of the basic moieties, in addition to the XPS analyses, an experimental degradation test at pH 3.0 was performed. Thereby, the treatment of MO by the ZY/PMS system at pH 3.0 was carried out and the results are shown in Fig. S3. It was found that the pollutant removal by the ZY/PMS system was lower at pH 3.0. This acidic pH allowed us to protonate some of the basic moieties on the zeolite (Munthali et al. 2014), thus limiting the initial proton transfer from PMS (Eq. 1). All the above results support the PMS activation mechanism summarized by Eqs. 1–3. However, we also suggest developing further research to better clarify the superficial interactions between ZY and PMS using experimental techniques such as in situ ATR-FTIR and/or theoretical calculations about electronic density difference (EDD) analysis and transition state (TS) via molecular dynamics simulations (Tang et al. 2023, 2024).

The role of singlet oxygen in the CIP elimination is also evidenced in the formation of the two transformation products (Fig. 4A). These two primary by-products could come from the attack of the singlet oxygen to the piperazyl moiety on CIP. The reactivity of the piperazyl moiety is also supported by NBO analyses (Fig. 4B), where it is shown that such functional group of CIP had the highest negative net charges, denoting that the piperazyl moiety is very reactive toward electrophilic reactive species such as singlet oxygen. Indeed, it is proposed that the P1 and P2 formations begin through an electron transfer from the piperazyl moiety to ^1^O_2_ (Clennan and Pace 2005; Liang and Su 2009; Matzek and Carter 2016; Salma et al. 2016). The singlet oxygen attacks the tertiary amine on the piperazyl ring leading to a cation radical species that reacts with water, forming a secondary hydroxylamine. This hydroxylamine evolves to a di-imine intermediate; and afterward, water can promote di-imine hydrolysis yielding P1 (Dennis et al. 1967). Besides, from the attack of ^1^O_2_ to the secondary amine on the piperazyl ring, a cation radical is produced. The cation radical experiences an α-deprotonation (Hu et al. 2013), generating an intermediate alcohol. Afterward, such intermediate alcohol evolved into imine and aldehyde groups, and the subsequent imine hydrolysis yields P2.

Regarding the MO transformation by the ZY/PMS system, we can say that this dye also reacts with singlet oxygen and/or PMS inducing the cleavage of the central azo moiety (as supported by the UV-spectra in Fig. S4). The breakdown of the azo-benzene bond produces 4-diazonium-benzene-sulfonate and 4-dimethylaminophenol (DMAP). The diazonium-benzene compounds react with water, thus forming benzene-sulfonic acid and/or p-hydroxy-benzene-sulfonic acid, having absorption at wavelengths below 280 nm. In turn, DMAP may also be oxidized by singlet oxygen or PMS forming the p-nitrophenol product that has a typical UV-light absorption band centered at ∼318 nm (as shown in Fig. S4) (Nihemaiti et al. 2020; Lee et al. 2020; Serna-Galvis et al. 2023a).

Also, it is important to mention that the reuse test for the MO treatment was carried out (Fig. S5). It was found the ZY/PMS system achieved degradations higher than 90% of the dye, even after the third cycle of reuse. The high reusability of the zeolite is supported by the involved mechanism (Eqs. 1–3), which comprises a proton transfer, as the initial step of the PMS activation; and in such a route, the surface is not modified significantly. Therefore, ZY remains useful during the three cycles to activate PMS toward the production of ^1^O_2_ for degrading MO. Besides, the high reusability also is an indirect indicator of the ZY stability during the catalytic process. On the other side, we should mention that the identification of optimal operational conditions in future work, by applying machine learning models along with response surface methodology (Sheikhmohammadi et al. 2024), could maximize the pollutant degradation and the zeolite performance for several reuse cycles.

### Treatment extent: biodegradability, antibiotic activity removal, matrix effects, and comparison with other catalytic systems

Considering the highest capability of ZY to activate PMS for CIP degradation, the extent of the ZY/PMS system for the pollutant treatment was assessed. We must mention that singlet oxygen has a low mineralizing capability, i.e., total organic carbon-TOC removal, because this ROS typically has lower reactivity toward the by-products compared to the parent compounds (Paredes-Laverde et al. 2023). This contrasts with processes that form non-selective radical species such as hydroxyl radical or sulfate radical, which are mineralizing systems (He et al. 2014; Hinojosa Guerra et al. 2019). Then, for the treatment extent of processes based on non-radical ROS (e.g., the ZY/PMS), it is better to consider aspects such as biodegradability, toxicity, antibiotic activity removal, and matrix effects; which were evaluated as detailed below.

The BiodegPred system was employed to predict biodegradability (related to the action of aerobic microorganisms) and toxicity to mammals (which can be acquired through exposure via ingestion of water polluted with pharmaceuticals or their transformation products). Then, predictions (based on the chemical structure) for the primary transformation products are presented in Table 2. According to the predictions, the primary degradation products (P1 and P2) would be non-ready biodegradable but they would have low toxicity. These by-products would have a low change in the biodegradability parameter regarding CIP due to the small structural modifications induced by the ZY/PMS process concerning the initial pharmaceutical. It is important to remark that these theoretical results are an initial approach (Garcia-Martin et al. 2020b) and future work should test experimentally the biodegradability and toxicity of the treated solution.

**Table 2:** Predicted biodegradability and toxicity to mammals for the primary transformation products coming from the CIP treatment by the ZY/PMS system

Compound	Biodegradability* (NITE)	Toxicity* (PPBD)
CIP	Non-ready biodegradable (reliability, 94.22%)	Low toxicity (reliability, 70.99%)
P1	Non-ready biodegradable (reliability, 94.14%)	Low toxicity (reliability, 71.05%)
P2	Non-ready biodegradable (reliability, 94.23%)	Low toxicity (reliability, 72.57%)

*Biodegradability and toxicity were predicted using a support vector machine (SVM), discriminating between two categories (i.e., “ready biodegradable” and “non-ready biodegradable” for the biodegradability parameter; or “low toxicity” and “high toxicity” for the toxicity parameter according to the reference (Garcia-Martin et al. 2020b))

**Fig. 5:**
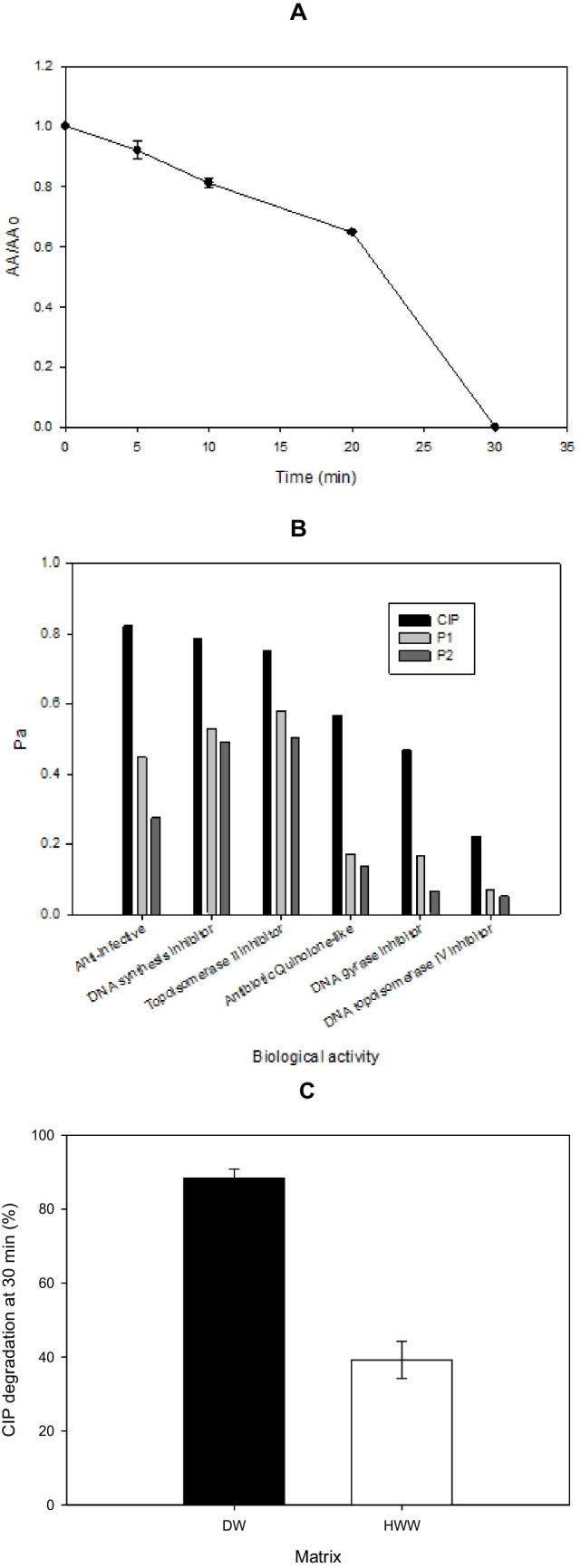
Extent of the CIP treatment by the ZY/PMS system. **A** Evolution of the antimicrobial activity (AA). **B** Theoretical calculations of biological activity for CIP and its by-products (*Pa, probability to be active). **C** Comparison of CIP degradation in distilled water (DW) and a complex matrix (simulated hospital wastewater (HWW), the composition is detailed in Table S3). *Experimental conditions:* [ZY] = 0.2 g L^−1^, [PMS] = 500 µmol L^−1^, and [CIP] = 30.6 µmol L^−^^1^. *Note:* the predictions of the biological activity were carried out on the free online version of PASS software (W2D Team - PharmaExpert 2021)

On the other hand, to analyze the risk of the resultant solution to the proliferation of antibiotic-resistant microorganisms, the experimental evolution of antimicrobial activity (AA) during the treatment was followed. The process led to a significant decrease in AA after 30 min of treatment (Fig. 5A). The decrease in AA is related to the primary transformations induced on CIP by the ZY/PMS system (Serna-Galvis et al. 2023a). The treatment changed the piperazyl ring structure of the target pollutant (Fig. 4A), which controls the antibacterial potency and efflux inhibition (Andersson and MacGowan 2003). Besides, the piperazyl ring opening (P1) and ring cleavage plus oxidation (P2) can modify the acid/base speciation and decrease the lipophilicity, diminishing cell permeability (Paul et al. 2010) and the antibiotic binding to bacterial DNA topoisomerases or DNA gyrase (Alovero et al. 2000). Thereby, the AA is decreased. The AA results were also reinforced by theoretical analyses about the probability of being active (Pa) for the transformation products. Pa values for biological activities such as anti-infective substance, antibiotic quinolone-like, DNA synthesis inhibitor, DNA topoisomerase IV inhibitor, DNA gyrase inhibitor, and topoisomerase II inhibitor (which are known as the main antimicrobial action mechanisms of CIP (DrugBank 2023)) were calculated. Figure 5B summarizes the Pa numbers for CIP and the primary products. It can be remarked that all the Pa values for the transformation products were lower than the corresponding numbers for the parent antibiotic, thus supporting the diminution in the AA after the treatment as observed in Fig. 5A.

In addition to the analyses of toxicity, biodegradability, and antimicrobial activity, the degrading action of the ZY/PMS process on CIP in simulated hospital wastewater (HWW, composition presented in Table S3) was assessed and compared to the process action on the pollutant in distilled water (DW). The degradation of the antibiotic in HWW after 30 min of treatment was ~ 40%. From Fig. 5C, it can also be noted that the degradation in the complex matrix was lower than in DW, which is linked to the interfering effects of the HWW components. For instance, urea can quench singlet oxygen (Michaeli and Feitelson 1995), affecting CIP degradation. Also, cations, such as Mg^2+^, Ca^2+^, Na^+^, K^+^, or NH_4_^+^, can interact with the ZY surface (Neddenriep 1968; Tahraoui et al. 2020), decreasing the activation of PMS (Eqs. 1 and 2), and consequently, this diminishes the CIP elimination in HWW regarding DW.

Finally, a comparison of our ZY/PMS system (for degrading CIP) with previous works in the literature was done. Table 3 presents such a comparison. It can be noted that our catalytic process has a superior capability to degrade the target antibiotic than its corresponding subsystems, in an analogous way to the UV/hematite/sulfite, UV/TiO_2_, or mixed metal oxides/PMS systems (Salma et al. 2016; Hu et al. 2020; Serna-Galvis et al. 2023b; Sheikhmohammadi et al. 2023). In contrast to the other processes mentioned in Table 3, our catalytic system degraded the target pollutant by attacks of a non-radical species. However, similar to the UV/hematite/sulfite (Sheikhmohammadi et al. 2023), in the ZY/PMS system, the matrix component reduces the efficiency of CIP degradation. Furthermore, the process based on the zeolite plus peroxymonosulfate leads to primary transformations of the piperazyl ring on CIP and decreases the antimicrobial activity of the treated samples, as also reported for UV/TiO_2_, or mixed metal oxides/PMS systems (Salma et al. 2016; Hu et al. 2020; Serna-Galvis et al. 2023b). Therefore, the comparison with other catalytic systems revealed that the ZY/PMS process can be an alternative to other catalytic AOPs for degrading recalcitrant antibiotics such as ciprofloxacin.

**Table 3:** Comparison of the CIP degradation by diverse heterogeneous catalytic systems

Degradation system	Main results	References
UV/hematite (α-Fe_2_O_3_)/sulfite	-Higher removal of CIP compared with their control subsystems (i.e., UV alone, UV/hematite, and UV/sulfite processes) -The main degradation mechanism involved hydroxyl and sulfate radicals -The presence of anions (Cl^−^, SO_4_^2−^, NO_3_^−^ and HCO_3_^−^) in the reaction medium decreased the antibiotic degradation	(Sheikhmohammadi et al. 2023)
UV/TiO_2_	-Significant direct photodegradation of CIP is obtained when UVC (254 nm) is used. However, antibiotic degradation is superior by using the UV/TiO_2_ process -The degradation routes involve direct photolysis and attack ^1^O_2_, O_2_•^−^, and HO•, generated in the photocatalytic system -The process leads to primary transformations on the piperazyl ring and C-F bond mainly -The TiO_2_-photocatalytic processes are able to decrease the AA associated with CIP	(Salma et al. 2016; Hu et al. 2020)
Mixed oxides of Co, Cu, Cr/PMS	-Low adsorption of CIP on the catalyst, low oxidation by PMS, and faster degradation by the catalyst/PMS combination -Sulfate radicals played the main degrading role in the catalytic process -Several primary transformation products from attacks to the piperazyl ring and C-F bond -Good performance of the catalytic process in the HWW matrix (close to the obtained in DW)	(Serna-Galvis et al. 2023b)
ZY/PMS	-Superior degradation of CIP by the catalytic system compared to the control subsystems (i.e., ZY or PMS acting alone) -Main degradation pathway: attacks of singlet oxygen -Primary transformation products revealed modifications on the piperazyl ring -The ZY/PMS decreased the AA associated with CIP -Degradation in a complex matrix (HWW) but slower than in distilled water	This work

## Conclusions

Zeolites having diverse structures and compositions were tested, showing that those materials with low Si/Al ratio presented the highest capability for the activation of PMS toward CIP degradation. Thereby, we can inform that Si/Al is a key factor that determines the presence of basic groups in the zeolite structure, which can activate PMS toward singlet oxygen. In the case of ZY, which showed the best degrading performance, it predominated the non-radical pathway, mainly the action of singlet oxygen. The singlet oxygen led to transformations on the piperazyl ring on CIP and this was consistent with the attacks of electro-rich moieties on the target pharmaceutical (according to NBO analyses). The zeolite/PMS process produced non-toxic substances from the CIP degradation and decreased the antimicrobial activity of the treated solution. In comparison with CIP, its by-products had lower biological activities, according to the theoretical predictions carried out in this work. Finally, it is important to mention that the ZY/PMS systems achieved a partial degradation of CIP in HWW, which was lower than in distilled water due to some competence of the matrix components by the singlet oxygen and/or a blocking of the active sites on the zeolite. Additionally, the comparison (in terms of intrinsic abilities of the processes) with other catalytic systems indicated that the ZY/PMS process can be an interesting option to other catalytic AOPs for degrading recalcitrant antibiotics such as ciprofloxacin.

## Supplementary Information

Below is the link to the electronic supplementary material.

Supplementary file1 (DOCX 499 KB)

## Data Availability

Data and materials will be available upon request to the authors.

## References

[CR1] Alovero FL, Pan X, Morris JE, Manzo RH, Fisher LM (2000) Engineering the specificity of antibacterial fluoroquinolones: benzenesulfonamide modifications at C-7 of ciprofloxacin change its primary target in Streptococcus pneumoniae from topoisomerase IV to gyrase. Antimicrob Agents Chemother 44:320–325. 10.1128/AAC.44.2.320-325.2000 10639357 10.1128/aac.44.2.320-325.2000 PMC89678

[CR2] Andersson MI, MacGowan AP (2003) Development of the quinolones. J Antimicrob Chemother 51(Suppl 1):1–11. 10.1093/jac/dkg212 10.1093/jac/dkg212 12702698

[CR3] Botero-Coy AM, Martínez-Pachón D, Boix C, Rincón RJ, Castillo N, Arias-Marín LP, Manrique-Losada L, Torres-Palma RA, Moncayo-Lasso A, Hernández F (2018) An investigation into the occurrence and removal of pharmaceuticals in Colombian wastewater. Sci Total Environ 642:842–853. 10.1016/j.scitotenv.2018.06.088 30045524 10.1016/j.scitotenv.2018.06.088

[CR4] Braschi I, Blasioli S, Gigli L, Gessa CE, Alberti A, Martucci A (2010) Removal of sulfonamide antibiotics from water: evidence of adsorption into an organophilic zeolite Y by its structural modifications. J Hazard Mater 178:218–225. 10.1016/j.jhazmat.2010.01.066 20133061 10.1016/j.jhazmat.2010.01.066

[CR5] Bunting SY, Lapworth DJ, Crane EJ, Grima-Olmedo J, Koroša A, Kuczyńska A, Mali N, Rosenqvist L, van Vliet ME, Togola A, Lopez B (2021) Emerging organic compounds in European groundwater. Environ Pollut 269:115945. 10.1016/j.envpol.2020.115945 33261962 10.1016/j.envpol.2020.115945

[CR6] Cataldo E, Salvi L, Paoli F, Fucile M, Masciandaro G, Manzi D, Masini CM, Mattii GB (2021) Application of zeolites in agriculture and other potential uses: a review. Agronomy 11:1–14

[CR7] Chen D, Bai Q, Ma T, Jing X, Tian Y, Zhao R, Zhu G (2022) Stable metal–organic framework fixing within zeolite beads for effectively static and continuous flow degradation of tetracycline by peroxymonosulfate activation. Chem Eng J 435:134916. 10.1016/j.cej.2022.134916

[CR8] Clennan EL, Pace A (2005) Advances in singlet oxygen chemistry. Tetrahedron 61:6665–6691. 10.1016/j.tet.2005.04.017

[CR9] Dai Y, Peng Q, Liu K, Tang X, Zhou M, Jiang K, Zhu B (2021) Activation of peroxymonosulfate by chrysotile to degrade dyes in water: performance enhancement and activation mechanism. Minerals 11:1–18. 10.3390/min11040400

[CR10] Decision 2020/1161/EU (2020) Commision Implementing Decision (EU) 2020/1161-4 August 2020-establishing a watch list of substances for Union-wide monitoring in the field of water policy pursuant to Directive 2008/105/EC of the European Parliament and of the Council. Off J Eur Union 257:32–35

[CR11] Dennis W, Hull L, Rosenblatt D (1967) Oxidations of amines IV oxidative fragmentation. J Org Chem 32:3783–3787

[CR12] DrugBank (2023) Ciprofloxacin. https://go.drugbank.com/drugs/DB00537. Accessed 15 Jul 2023

[CR13] Elmaadawy K, Houa H, Hua J, Liu B (2021) Molecular sieve 4A assisted peroxymonosulfate activation for humic acid degradation as a model of persistent organic matter in landfill leachate. Solid State Technol 64:8557

[CR14] Feng C, Han W, Deng Y, Zhang B, Zhao X, Han D (2021) Key technology and application analysis of zeolite adsorption for energy storage and heat-mass transfer process: a review. Renew Sustain Energy Rev 144:110954. 10.1016/j.rser.2021.110954

[CR15] Fowkes AJ, Ibberson RM, Rosseinsky MJ (2002) Structural characterization of the redox behavior in copper-exchanged sodium zeolite Y by high-resolution powder neutron diffraction. Chem Mater 14:590–602. 10.1021/cm010504b

[CR16] Fu X, Zhang J, Zhao H, Zhang S, Nie T, Zhang Y, Lu J (2020) Enhanced peroxymonosulfate activation by coupling zeolite-supported nano-zero-valent iron with weak magnetic field. Sep Purif Technol 230:115886. 10.1016/j.seppur.2019.115886

[CR17] Garcia-Martin JA, Chavarria M, de Lorenzo V, Pazos F (2020a) SVM Biodegradability predictor. In: BiodegPred. https://sysbiol.cnb.csic.es/BiodegPred/. Accessed 10 Sep 2023

[CR18] Garcia-Martin JA, Chavarriá M, De Lorenzo V, Pazos F (2020b) Concomitant prediction of environmental fate and toxicity of chemical compounds. Biol Methods Protoc 5:1–10. 10.1093/biomethods/bpaa025 10.1093/biomethods/bpaa025 PMC7750720 33376807

[CR19] Guan C, Jiang J, Pang S, Luo C, Ma J, Zhou Y, Yang Y (2017) Oxidation kinetics of bromophenols by nonradical activation of peroxydisulfate in the presence of carbon nanotube and formation of brominated polymeric products. Environ Sci Technol 51:10718–10728. 10.1021/acs.est.7b02271 28806069 10.1021/acs.est.7b02271

[CR20] He X, Shea KEO (2020) Rapid transformation of H1-antihistamines cetirizine (CET) and diphenhydramine (DPH) by direct peroxymonosulfate (PMS) oxidation. J Hazard Mater 398:123219. 10.1016/j.jhazmat.2020.123219 32768849 10.1016/j.jhazmat.2020.123219

[CR21] He X, Mezyk SP, Michael I, Fatta-Kassinos D, Dionysiou DD (2014) Degradation kinetics and mechanism of β-lactam antibiotics by the activation of H2O2 and Na2S2O8 under UV-254nm irradiation. J Hazard Mater 279:375–383. 10.1016/j.jhazmat.2014.07.008 25086235 10.1016/j.jhazmat.2014.07.008

[CR22] Hinojosa Guerra MM, Oller Alberola I, Malato Rodriguez S, Agüera López A, Acevedo Merino A, Quiroga Alonso JM (2019) Oxidation mechanisms of amoxicillin and paracetamol in the photo-Fenton solar process. Water Res 156:232–240. 10.1016/j.watres.2019.02.055 30921539 10.1016/j.watres.2019.02.055

[CR23] Hu J, Wang J, Nguyen TH, Zheng N (2013) The chemistry of amine radical cations produced by visible light photoredox catalysis. Beilstein J Org Chem 9:1977–2001. 10.3762/bjoc.9.234 24204409 10.3762/bjoc.9.234 PMC3817571

[CR24] Hu X, Hu X, Peng Q, Zhou L, Tan X, Jiang L, Tang C, Wang H, Liu S, Wang Y, Ning Z (2020) Mechanisms underlying the photocatalytic degradation pathway of ciprofloxacin with heterogeneous TiO2. Chem Eng J 380:122366. 10.1016/j.cej.2019.122366

[CR25] Jiang N, Shang R, Heijman SGJ, Rietveld LC (2018) High-silica zeolites for adsorption of organic micro-pollutants in water treatment: a review. Water Res 144:145–161. 10.1016/j.watres.2018.07.017 30025266 10.1016/j.watres.2018.07.017

[CR26] Kenvin J, Mitchell S, Sterling M, Warringham R, Keller TC, Crivelli P, Jagiello J, Pérez-Ramírez J (2016) Quantifying the complex pore architecture of hierarchical faujasite zeolites and the impact on diffusion. Adv Funct Mater 26:5621–5630. 10.1002/adfm.201601748

[CR27] Khaleque A, Alam MM, Hoque M, Mondal S, Haider JB, Xu B, Johir MAH, Karmakar AK, Zhou JL, Ahmed MB, Moni MA (2020) Zeolite synthesis from low-cost materials and environmental applications: a review. Environ Adv 2:100019. 10.1016/j.envadv.2020.100019

[CR28] Kiejza D, Kotowska U, Poli W, Karpi J (2021) Peracids - new oxidants in advanced oxidation processes: the use of peracetic acid, peroxymonosulfate, and persulfate salts in the removal of organic micropollutants of emerging concern − a review. Sci Total Environ 790:148195. 10.1016/j.scitotenv.2021.148195 34380254 10.1016/j.scitotenv.2021.148195

[CR29] Kong L, Fang G, Xi X, Wen Y, Chen Y, Xie M, Zhu F, Zhou D, Zhan J (2021) A novel peroxymonosulfate activation process by periclase for efficient singlet oxygen-mediated degradation of organic pollutants. Chem Eng J 403:126445. 10.1016/j.cej.2020.126445

[CR30] Lee J, von Gunten U, Kim J (2020) Persulfate-based advanced oxidation: critical assessment of opportunities and roadblocks. Environ Sci Technol 54:3064–3081. 10.1021/acs.est.9b07082 32062964 10.1021/acs.est.9b07082

[CR31] Li C, Huang Y, Dong X, Sun Z, Duan X, Ren B, Zheng S, Dionysiou DD (2019) Highly efficient activation of peroxymonosulfate by natural negatively-charged kaolinite with abundant hydroxyl groups for the degradation of atrazine. Appl Catal B Environ 247:10–23. 10.1016/j.apcatb.2019.01.079

[CR32] Li J, Gao M, Yan W, Yu J (2022) Regulation of the Si/Al ratios and Al distributions of zeolites and their impact on properties. Chem Sci 14:1935–1959. 10.1039/d2sc06010h 36845940 10.1039/d2sc06010h PMC9945477

[CR33] Liang C, Su HW (2009) Identification of sulfate and hydroxyl radicals in thermally activated persulfate. Ind Eng Chem Res 48:5558–5562. 10.1021/ie9002848

[CR34] Liu L, Li Y, Pang Y, Lan Y, Zhou L (2020) Activation of peroxymonosulfate with CuCo2O4@kaolin for the efficient degradation of phenacetin. Chem Eng J 401:126014. 10.1016/j.cej.2020.126014

[CR35] Manrique C, Guzmán A, Pérez-Pariente J, Márquez-Álvarez C, Echavarría A (2016) Effect of synthesis conditions on zeolite beta properties and its performance in vacuum gas oil hydrocracking activity. Microporous Mesoporous Mater 234:347–360. 10.1016/j.micromeso.2016.07.017

[CR36] Martínez-Iñesta MM, Peral I, Proffen T, Lobo RF (2005) A pair distribution function analysis of zeolite beta. Microporous Mesoporous Mater 77:55–66. 10.1016/j.micromeso.2004.07.040

[CR37] Matzek LW, Carter KE (2016) Activated persulfate for organic chemical degradation: a review. Chemosphere 151:178–188. 10.1016/j.chemosphere.2016.02.055 26938680 10.1016/j.chemosphere.2016.02.055

[CR38] Mendoza C, Echavarría A (2022) A systematic study on the synthesis of nanosized Y zeolite without using organic structure-directing agents: control of Si/Al ratio. J Porous Mater 29:907–919. 10.1007/s10934-022-01218-0

[CR39] Michaeli A, Feitelson J (1995) Reactivity of singlet oxygen toward large peptides. Photochem Photobiol 61:255–260. 10.1111/j.1751-1097.1995.tb03968.x 7716187 10.1111/j.1751-1097.1995.tb03968.x

[CR40] Munthali MW, Elsheikh MA, Johan E, Matsue N (2014) Proton adsorption selectivity of zeolites in aqueous media: effect of Si/Al ratio of zeolites. Molecules 19:20468–20481. 10.3390/molecules191220468 25493632 10.3390/molecules191220468 PMC6271039

[CR41] Neddenriep RJ (1968) Sodium cation adsorption sites in zeolite types X and Y. J Colloid Interface Sci 28:293–304. 10.1016/0021-9797(68)90133-1

[CR42] Nihemaiti M, Permala RR, Croué JP (2020) Reactivity of unactivated peroxymonosulfate with nitrogenous compounds. Water Res 169:115221. 10.1016/j.watres.2019.115221 31678752 10.1016/j.watres.2019.115221

[CR43] Oliveira TS, Aukidy M Al, Verlicchi P (2018) Occurrence of common pollutants and pharmaceuticals in hospital effluents. In: Verlicchi P (ed) Hospital wastewaters - characteristics, management, treatment and environmental risks. Springer International Publishing, pp 17–32

[CR44] Otomo R, Osuga R, Kondo JN, Kamiya Y, Yokoi T (2019) Cs-Beta with an Al-rich composition as a highly active base catalyst for Knoevenagel condensation. Appl Catal A Gen 575:20–24. 10.1016/j.apcata.2019.02.014

[CR45] Pandey A, Pathak VM, Navneet RM (2024) A feasible approach for azo-dye (methyl orange) degradation by textile effluent isolate Serratia marcescens ED1 strain for water sustainability: AST identification, degradation optimization and pathway hypothesis. Heliyon 10:e32339. 10.1016/j.heliyon.2024.e32339 38961949 10.1016/j.heliyon.2024.e32339 PMC11219335

[CR46] Paredes-Laverde M, Porras J, Acelas N, Romero-Hernández JJ, Jojoa-Sierra SD, Huerta L, Serna-Galvis EA, Torres-Palma RA (2023) Rice husk–based pyrogenic carbonaceous material efficiently promoted peroxymonosulfate activation toward the non-radical pathway for the degradation of pharmaceuticals in water. Environ Sci Pollut Res 30:123616–123632. 10.1007/s11356-023-30785-1 10.1007/s11356-023-30785-1 PMC10746782 37991611

[CR47] Patel M, Kumar R, Kishor K, Mlsna T, Pittman CU, Mohan D (2019) Pharmaceuticals of emerging concern in aquatic systems: chemistry, occurrence, effects, and removal methods. Chem Rev 119:3510–3673. 10.1021/acs.chemrev.8b00299 30830758 10.1021/acs.chemrev.8b00299

[CR48] Paul T, Dodd MC, Strathmann TJ (2010) Photolytic and photocatalytic decomposition of aqueous ciprofloxacin: transformation products and residual antibacterial activity. Water Res 44:3121–3132. 10.1016/j.watres.2010.03.002 20363011 10.1016/j.watres.2010.03.002

[CR49] Raghavachari K (2001) Theoretical chemistry accounts: new century issue. Theoretical chemistry accounts. Springer, Berlin Heidelberg, Berlin, Heidelberg, pp 361–363

[CR50] Rajaei H, Esmaeilzadeh F, Mowla D (2021) Elucidation of Si/Al ratio on physicochemical properties of HZSM-5 zeolites. J Therm Anal Calorim 146:581–586. 10.1007/s10973-020-09993-1

[CR51] Salma A, Thoröe-Boveleth S, Schmidt TC, Tuerk J (2016) Dependence of transformation product formation on pH during photolytic and photocatalytic degradation of ciprofloxacin. J Hazard Mater 313:49–59. 10.1016/j.jhazmat.2016.03.010 27054664 10.1016/j.jhazmat.2016.03.010

[CR52] Sbardella L, Velo I, Comas J (2020) Integrated assessment of sulfate-based AOPs for pharmaceutical active compound removal from wastewater. J Clean Prod 260:121014. 10.1016/j.jclepro.2020.121014

[CR53] Schoonheydt RA, Geerlings P, Pidko EA, van Santen RA (2012) The framework basicity of zeolites. J Mater Chem 22:18705–18717. 10.1039/C2JM31366A

[CR54] Serna-Galvis EA, Martínez-Mena YL, Arboleda-Echavarría J, Hoyos-Ayala DA, Echavarría-Isaza A, Torres-Palma RA (2023a) Zeolite 4A activates peroxymonosulfate toward the production of singlet oxygen for the selective degradation of organic pollutants. Chem Eng Res des 193:121–131. 10.1016/j.cherd.2023.03.015

[CR55] Serna-Galvis EA, Mendoza-Merlano C, Torres-Palma RA, Echavarría-Isaza A, Hoyos-Ayala DA (2023) Materials based on Co, Cu, and Cr as activators of PMS for degrading a representative antibiotic—the strategy for utilization in water treatment and warnings on metal leaching. Molecules 28:4536. 10.3390/molecules28114536 37299012 10.3390/molecules28114536 PMC10254359

[CR56] Sheikhmohammadi A, Asgari E, Manshouri M (2021) The synergistic effect of O3 and H2O2 on the butyl p-hydroxybenzoate photo-catalytic degradability by UVC/ZnO: Efficiency, kinetic, pathway, mechanism. Optik (Stuttg) 239:166673. 10.1016/j.ijleo.2021.166673

[CR57] Sheikhmohammadi A, Asgari E, Alinejad N, Hashemzadeh B (2023) Photocatalytic oxidation of ciprofloxacin by UV/ α-Fe2O3/sulfite: mechanism, kinetic, degradation pathway. Int J Environ Health Res 33:192–205. 10.1080/09603123.2021.2013453 34878341 10.1080/09603123.2021.2013453

[CR58] Sheikhmohammadi A, Alamgholiloo H, Golaki M, Khakzad P, Asgari E, Rahimlu F (2024) Cefixime removal via WO3/Co-ZIF nanocomposite using machine learning methods. Sci Rep 14:13840. 10.1038/s41598-024-64790-2 38879660 10.1038/s41598-024-64790-2 PMC11180210

[CR59] Sidhu H, Bae H, Ogram A, Connor GO (2021) Azithromycin and ciprofloxacin can promote antibiotic. Appl Environ Microbiol 87:1–15 10.1128/AEM.00373-21 PMC8315170 34085858

[CR60] Sun X, Xu D, Dai P, Liu X, Tan F, Guo Q (2020) Efficient degradation of methyl orange in water via both radical and non-radical pathways using Fe-Co bimetal-doped MCM-41 as peroxymonosulfate activator. Chem Eng J 402:125881. 10.1016/j.cej.2020.125881

[CR61] Szabó L, Tóth T, Engelhardt T, Rácz G, Mohácsi-Farkas C, Takács E, Wojnárovits L (2016) Change in hydrophilicity of penicillins during advanced oxidation by radiolytically generated •OH compromises the elimination of selective pressure on bacterial strains. Sci Total Environ 551–552:393–403. 10.1016/j.scitotenv.2016.02.002 10.1016/j.scitotenv.2016.02.002 26881730

[CR62] Tahraoui Z, Nouali H, Marichal C, Forler P, Klein J, Daou TJ (2020) Influence of the compensating cation nature on the water adsorption properties of zeolites. Molecules 25:944. 10.3390/molecules25040944 32093246 10.3390/molecules25040944 PMC7070582

[CR63] Tang M, Wan J, Wang Y, Ye G, Yan Z, Ma Y, Sun J (2023) Insights into molecular imprinting polydopamine in-situ activating peroxydisulfate for targeted removal of refractory organic pollutants: overlooked N site. Appl Catal B Environ 334:122852. 10.1016/j.apcatb.2023.122852

[CR64] Tang M, Wan J, Wang Y, Ye G, Yan Z, Ma Y, Sun J (2024) Overlooked role of void-nanoconfined effect in emerging pollutant degradation: modulating the electronic structure of active sites to accelerate catalytic oxidation. Water Res 249:120950. 10.1016/j.watres.2023.120950 38056201 10.1016/j.watres.2023.120950

[CR65] Tomasi J, Mennucci B, Cammi R (2005) Quantum mechanical continuum solvation models. Chem Rev 105:2999–3094. 10.1021/cr9904009 16092826 10.1021/cr9904009

[CR66] Ushani U, Lu X, Wang J, Zhang Z, Dai J, Tan Y, Wang S, Li W, Niu C, Cai T, Wang N, Zhen G (2020) Sulfate radicals-based advanced oxidation technology in various environmental remediation: a state-of-the–art review. Chem Eng J 402:126232. 10.1016/j.cej.2020.126232

[CR67] Verlicchi P, Aukidy MA, Zambello E (2015) What have we learned from worldwide experiences on the management and treatment of hospital effluent? — An overview and a discussion on perspectives. Sci Total Environ 514:467–491. 10.1016/j.scitotenv.2015.02.020 25698384 10.1016/j.scitotenv.2015.02.020 PMC7112026

[CR68] W2D Team - PharmaExpert (2021) PASS online. http://www.pharmaexpert.ru/passonline/index.php. Accessed 2 May 2021

[CR69] Wang W, Chen M, Wang D, Yan M, Liu Z (2021) Different activation methods in sulfate radical-based oxidation for organic pollutants degradation: catalytic mechanism and toxicity assessment of degradation intermediates. Sci Total Environ 772:145522. 10.1016/j.scitotenv.2021.145522 33571779 10.1016/j.scitotenv.2021.145522

[CR70] Wang Q, Xu Z, Jiang Y, Lu J, Li H, Du X, Wang Z (2022) Efficient peroxymonosulfate activation and less metallic leaching through kaolin@MnCo2O4 for bisphenol A degradation in environmental remediation. Appl Surf Sci 585:152705. 10.1016/j.apsusc.2022.152705

[CR71] Xiao G, Xu T, Faheem M, Xi Y, Zhou T, Moryani HT, Bao J, Du J (2021) Evolution of singlet oxygen by activating peroxydisulfate and peroxymonosulfate: a review. Int J Environ Res Public Health 18:3344. 10.3390/ijerph18073344 33804931 10.3390/ijerph18073344 PMC8036714

[CR72] Xu X, Zhang Y, Zhou S, Huang R, Huang S, Kuang H, Zeng X, Zhao S (2021) Activation of persulfate by MnOOH: degradation of organic compounds by nonradical mechanism. Chemosphere 272:129629. 10.1016/j.chemosphere.2021.129629 33486458 10.1016/j.chemosphere.2021.129629

[CR73] Zeolyst International (2015) Zeolite Beta (CP814C*). 1–6

[CR74] Zhou Y, Gao Y, Pang S, Jiang J, Yang Y, Ma J, Yang Y (2018) Oxidation of fluoroquinolone antibiotics by peroxymonosulfate without activation: kinetics, products, and antibacterial deactivation. Water Res 145:210–219. 10.1016/j.watres.2018.08.026 30142519 10.1016/j.watres.2018.08.026

