# Supplementary material for: Comparison of three different zeolites to activate peroxymonosulfate for the degradation of the pharmaceutical ciprofloxacin in water
Source: Environ Sci Pollut Res Int. 2025 Feb 28;32(11):6856–70. doi: 10.1007/s11356-025-35994-4 (PMC11928394; doi:10.1007/s11356-025-35994-4)
Supplement: Supplementary file 1 — Supplementary file1 (DOCX 499 KB) [file 11356_2025_35994_MOESM1_ESM.docx]

**Supplementary Material**

**Comparison of three different zeolites to activate peroxymonosulfate for the degradation of the pharmaceutical ciprofloxacin in water**

Efraím A. Serna-Galvis^a,b^, Carlos Mendoza-Merlano^b^, Johana Arboleda-Echavarría^b,c^, Ricardo A. Torres-Palma^a^, Adriana Echavarría-Isaza^b,*^

*^a^ Grupo de Investigación en Remediación Ambiental y Biocatálisis (GIRAB), Instituto de Química, Facultad de Ciencias Exactas y Naturales, Universidad de Antioquia UdeA, Calle 70 # 52-21, Medellín, Colombia*

*^b^ Grupo de Catalizadores y Adsorbentes (CATALAD), Instituto de Química, Facultad de Ciencias Exactas y Naturales, Universidad de Antioquia UdeA, Calle 70 # 52-21, Medellín, Colombia*

*^c^ Escuela de Microbiología, Universidad de Antioquia UdeA, Calle 70 # 52-21, Medellín, Colombia*

**Corresponding author:* [adriana.echavarria@udea.edu.co](mailto:adriana.echavarria@udea.edu.co)

**Supplementary figures**

**A**

**B**

**C**

**Fig. S1.** Determination of the basicity of the considered zeolites by CO_2_ adsorption (TPD-CO_2_ results). **A.** ZY, **B.** ZB, and **C.** CP814C*.

**A**

**B**

**Fig. S2.** Structures of the target pollutants **A.** Methyl Orange (MO).

**B.** Ciprofloxacin (CIP) and its pKa_1_.

**Supplementary tables**

**Table S1.** Elemental composition of ZY.

| **Element** | **Si** | **Al** | **Na** | **O** |
| --- | --- | --- | --- | --- |
| Weight Composition  (%) | 20.7 | 8.3 | 14.1 | 56.9 |

**Table S2.** Oxygen surface composition (percentage) for the basic groups on the ZY surface before and after interaction with PMS**.**

| **Zeolite*** | Al-O^-^ | Si-O^-^ |
| --- | --- | --- |
| ZY before interaction | 40.0 | 20.7 |
| ZY after  interaction | 33.4 | 8.8 |

*Before the interaction with PMS, the AlO^-^ and SiO^-^

moieties on ZY have Na^+^ as compensation cations.

**Fig. S3.** Degradation of MO by the ZY/PMS system at different initial pHs. *Experimental conditions:* [Zeolite]= 0.2 g L^-1^, [PMS]= 500 µmol L^-1^, and [MO]= 30.6 µmol L^-1^, pH nat: 5.6.

**Fig. S4.** Spectra of methyl orange solution without treatment (MO) and after 10 min of the treatment using the ZY/PMS system (MO treated by ZY/PMS).

**Fig. S5.** Reuse cycles for the MO treatment by ZY/PMS. *Experimental conditions:* [Z4A]= 0.2 g L^-1^, [PMS]= 500 µmol L^-1^, and [MO]= 30.6 µmol L^-1^.

**Table S3.** Composition of the simulated hospital wastewater (HWW).

| **Component** | **Concentration (mg L^-1^)** | | **Concentration (µmol L^-1^)** | | |
| --- | --- | --- | --- | --- | --- |
| NaCl | | 76 | | 1300 |  |
| NH_4_Cl | | 50 | | 940 |  |
| Na_2_SO_4_ | | 100.5 | | 710 |  |
| KCl | | 100 | | 1340 |  |
| KH_2_PO_4_ | | 50 | | 370 |  |
| CaCl_2_-2H_2_O | | 50 | | 340 |  |
| Urea | | 1260 | | 2100 |  |
| pH | | 6.5 | | |  |
